# A mixed methods process evaluation of a person-centred falls prevention program

**DOI:** 10.1186/s12913-019-4614-z

**Published:** 2019-11-28

**Authors:** Rebecca L. Morris, Keith D. Hill, Ilana N. Ackerman, Darshini Ayton, Glenn Arendts, Caroline Brand, Peter Cameron, Christopher D. Etherton-Beer, Leon Flicker, Anne-Marie Hill, Peter Hunter, Judy A. Lowthian, Renata Morello, Samuel R. Nyman, Julie Redfern, De Villiers Smit, Anna L. Barker

**Affiliations:** 1 School of Public Health and Preventive Medicine, Monash University, Melbourne, Australia; 2 Rehabilitation, Ageing and Independent Living (RAIL) Research Centre, Monash University, Melbourne, Australia; 3 School of Physiotherapy and Exercise Science, Curtin University, Perth, Australia; 4 University of Western Australia, Perth, Australia; 5 Harry Perkins Institute of Medical Research, Perth, Australia; 6 Melbourne EpiCentre, University of Melbourne and Melbourne Health, Melbourne, Australia; 7 Alfred Health, Melbourne, Australia; 8 Royal Perth Hospital, Perth, Australia; 9 Bolton Clarke Research Institute, Bolton Clarke, Melbourne, Australia; 10 Department of Psychology and Ageing & Dementia Research Centre, now at Department of Medical Science and Public Health, Bournemouth University, Dorset, UK; 11 University of Sydney, Westmead Applied Research Centre, Faculty of Medicine and Health, the George Institute for Global Health, Sydney, Australia

**Keywords:** Falls prevention, fractures, older adults, emergency department, process evaluation, complex intervention, mixed methods

## Abstract

**Background:**

RESPOND is a telephone-based falls prevention program for older people who present to a hospital emergency department (ED) with a fall. A randomised controlled trial (RCT) found RESPOND to be effective at reducing the rate of falls and fractures, compared with usual care, but not fall injuries or hospitalisations. This process evaluation aimed to determine whether RESPOND was implemented as planned, and identify implementation barriers and facilitators.

**Methods:**

A mixed-methods evaluation was conducted alongside the RCT. Evaluation participants were the RESPOND intervention group (*n* = 263) and the clinicians delivering RESPOND (*n* = 7). Evaluation data were collected from participant recruitment and intervention records, hospital administrative records, audio-recordings of intervention sessions, and participant questionnaires. The Rochester Participatory Decision-Making Scale (RPAD) was used to evaluate person-centredness (score range 0 (worst) - 9 (best)). Process factors were compared with pre-specified criteria to determine implementation fidelity. Six focus groups were held with participants (*n* = 41), and interviews were conducted with RESPOND clinicians (*n* = 6). Quantitative data were analysed descriptively and qualitative data thematically. Barriers and facilitators to implementation were mapped to the ‘Capability, Opportunity, Motivation – Behaviour’ (COM-B) behaviour change framework.

**Results:**

RESPOND was implemented at a lower dose than the planned 10 h over 6 months, with a median (IQR) of 2.9 h (2.1, 4). The majority (76%) of participants received their first intervention session within 1 month of hospital discharge with a median (IQR) of 18 (12, 30) days. Clinicians delivered the program in a person-centred manner with a median (IQR) RPAD score of 7 (6.5, 7.5) and 87% of questionnaire respondents were satisfied with the program. The reports from participants and clinicians suggested that implementation was facilitated by the use of positive and personally relevant health messages. Complex health and social issues were the main barriers to implementation.

**Conclusions:**

RESPOND was person-centred and reduced falls and fractures at a substantially lower dose, using fewer resources, than anticipated. However, the low dose delivered may account for the lack of effect on falls injuries and hospitalisations. The results from this evaluation provide detailed information to guide future implementation of RESPOND or similar programs.

**Trial registration:**

This study was registered with the Australian New Zealand Clinical Trials Registry, number ACTRN12614000336684 (27 March 2014).

## Background

Falls are the leading cause of hospital emergency department (ED) presentations for older people [1]. The evidence suggests that for fallers presenting to the ED, 13–33.3% will fall again within 6 months [2, 3], and 46–52% within 12 months [4, 5], highlighting the need for secondary falls prevention. In response to this clinical need, Barker et al. developed RESPOND: a falls prevention program targeting people presenting to ED with a fall to reduce their risk of subsequent falls (“*Respond to the first fall to prevent the second*”) [6, 7]. RESPOND was designed to include the characteristics that appear to distinguish successful falls prevention, and other behaviour change programs, from others: interventions delivered at sufficient dose; in a timely manner; incorporating person-centred education and goal setting; using a telephone-based motivational coaching approach [7]. A randomised controlled trial (RCT) of RESPOND showed the program to be effective at reducing the rate of falls and fractures, compared with usual care. There was no difference in fall injuries (other than fractures), or hospitalisation outcomes between groups [6].

RCTs are the gold standard for establishing the effectiveness of an intervention [8]. However, RCT results alone do not provide information related to what worked, how, and why. RESPOND is a complex intervention, comprising numerous potential “active ingredients” where the combination of components comprise more than the sum of its parts [9]. Process evaluations conducted alongside clinical trials can determine the degree of implementation fidelity, clarify causal mechanisms (how and why it worked), and identify contextual factors (barriers and facilitators) associated with outcomes [8]. This information can guide researchers, clinicians and policy makers to successfully implement similar programs in different settings [10].

To date, information related to process factors for falls prevention RCTs is limited. Of eleven RCTs of falls prevention programs targeting older adults who present to an ED with a fall [4, 5, 11–19], elements of process evaluation, such as reach, adherence and timeliness of program delivery are inconsistently reported. Only one program conducted a detailed process evaluation alongside the RCT [17, 20]. The evaluation attributed lack of program effectiveness to an insufficient number of referrals and recommendations resulting from medical assessments, and participants’ low compliance with advice [20]. No comprehensive process evaluation has been conducted on an RCT of a program that has been shown to reduce the rate of falls for older people who present to an ED with a fall, thus our understanding of critical success factors for reducing falls in this sub-optimally managed cohort remains limited. This process evaluation aimed to fill this gap in the literature by providing detailed insight into the RESPOND RCT results, and assist others in effectively translating the RESPOND program into real world settings, by addressing the following objectives:

1. To assess the degree to which RESPOND was implemented as planned; and
2. To identify barriers and facilitators to implementation from the perspectives of those delivering and receiving the intervention.

## Methods

### Study design

This paper reports a convergent parallel mixed-methods [21] process evaluation of the RESPOND RCT. Implementation fidelity is the degree to which an intervention is delivered as intended, and key components of evaluation of implementation fidelity have been variously categorised and defined [22]. For this study, components of implementation fidelity evaluated are: reach (the proportion of target cohort who participated in RESPOND); intervention participant adherence to minimum program requirements; RESPOND clinician adherence to key program components; and dose and timeliness of intervention delivered.

### Study setting and participants

A total of 541 community-dwelling adults aged 60–90 years, who had presented to one of two Australian public hospital EDs in Victoria and Western Australia with a fall, and had a planned discharge home within 72 h, were recruited to the RESPOND RCT. Exclusion criteria were: planned discharge to a residential aged care facility; current palliative care or terminal illness, requiring hands-on assistance to walk, non-English speaking, unable to use a telephone, a history of social aggression or psychosis, cognitive impairment (Mini Mental State Examination (MMSE) < 23) [23], or living > 50 km from the recruiting hospital. Recruited participants were randomised to either the RESPOND intervention or usual care and followed-up for 12 months. For those randomised to the intervention group, the first 6 months comprised the RESPOND program. RESPOND RCT details are published elsewhere [6, 7].

**Fig. 1:**
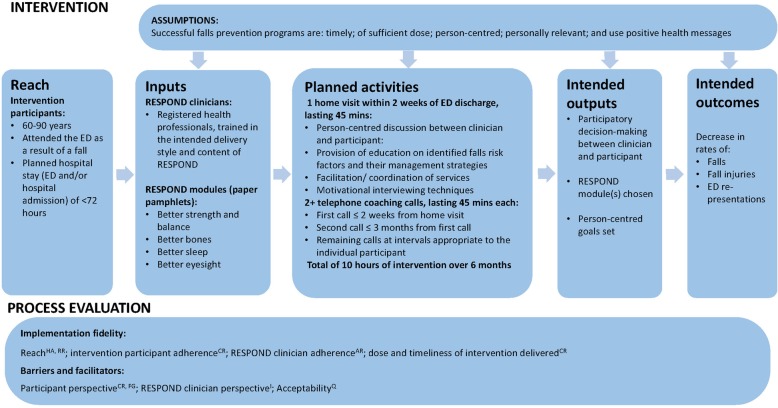
Key RESPOND intervention and process evaluation components. Process evaluation data sources: AR = audio-recordings of intervention sessions; CR = clinician records; FG = focus groups with intervention participants; HA = hospital administrative data; I = interviews with RESPOND clinicians; Q = intervention participant experience questionnaire; RR = recruitment records

RESPOND process evaluation participants were the trial intervention participants (*n* = 263) and the healthcare professionals delivering the program (*n* = 7: three physiotherapists, two occupational therapists, one dietitian, and one nurse). This process evaluation corresponds with the inputs, activities and outputs detailed in the RESPOND program logic model [24], and interrogates the assumptions underlying the model and the linkages between program components and trial outcomes (Fig. 1).

### RESPOND intervention

Intervention participants received an initial home visit from a RESPOND clinician. At this visit a falls risk assessment was conducted, using a valid and reliable tool: Falls Risk for Older People – Community setting (FROP-Com) [25, 26], and the RESPOND intervention was introduced. RESPOND consisted of four evidence-based modules related to falls risk factors: Better Strength and Balance; Better Bones; Better Eyesight; and Better Sleep. Each RESPOND module had an associated pamphlet with the slogan: “*Be Your Best*”. These each provided positively framed health messages related to the interventions such as: *“Exercise… can help you feel revitalised, relaxed and help you get a good night’s sleep”;* and *“With good eyesight you can…keep driving independently”.* Subsequent telephone coaching calls, using motivational interviewing approaches [27] were made by the RESPOND clinician over the 6 month intervention period. The timing, intended dose, and delivery style (person-centred education and goal setting, use of positive health messages, and motivational interviewing techniques), were pre-determined in the RCT protocol (summarised in Fig. 1) [7].

#### Clinician training

A standard operating procedures manual guided consistent delivery of program content and intended delivery style across the two sites. The lead clinician attended a motivational interviewing course, and provided face-to-face training to the other clinicians, using a ‘train the trainer’ approach [28]. RESPOND clinicians shadowed their senior during intervention sessions prior to commencing their own intervention delivery. The lead clinician held regular meetings with RESPOND clinicians to discuss specific issues or achievements with program delivery, present case studies, and provide trial updates.

### Data collection

#### Implementation fidelity

##### Reach

Program reach was evaluated through the number of participants recruited into the RCT compared to the number of potentially eligible patients presenting to the recruiting hospital EDs (identified from hospital administrative data). Reasons for declining to participate were coded. Recruitment data were collected by the RESPOND trial recruitment team and entered directly into a web based database via an iPad.

##### Intervention participant adherence

Participant adherence was defined as the proportion of participants who: i) had an initial home visit and at least two telephone coaching calls; ii) chose at least one RESPOND module to work through; and iii) set at least one goal. These data were recorded by the RESPOND clinicians in the project database.

##### RESPOND clinician adherence

RESPOND clinician adherence to key RESPOND components was evaluated through analysis of intervention session audio-recordings. The clinicians were initially asked to audio-record all intervention sessions, and part-way through the trial period this was changed to recording on a month on/ month off basis in order to reduce clinician burden. This component of the study evaluated whether the clinicians: delivered the intervention in the spirit of participatory decision-making, using motivational interviewing (MI) techniques; provided education related to falls risks and their management strategies; and provided linkage to appropriate local community health services. Examples of community linkage included referral to a strength and balance exercise group; seeking advice from their general practitioner (GP) regarding withdrawal of sedative medication or having a vitamin D test; or making an appointment with an optometrist for a vision test.

Motivational interviewing skills evaluated were: Open-ended questions, Affirmations (statements and gestures that recognise client strengths and acknowledge behaviours that lead in the direction of positive change); Reflections (listening to the participant and then making statements to demonstrate understanding); and Summaries (synopsis of the conversation) – “OARS” [29].

Education, community linkage and motivational interviewing were assessed as either being present (“1”) if there was an example of the clinician providing each component, or absent (“0”). Scoring guidelines were developed with definitions and examples for each component in order to assist with analysis.

Person-centeredness was analysed using the Rochester Participatory Decision-Making Scale (RPAD) [30]. This tool comprises nine aspects of participatory decision-making, each scoring “0” if no evidence of the item was present, “0.5” if some evidence, or a full point if strong evidence was present, with the exception of item 6, *‘Clinician’s medical language matches participant’s level of understanding’*, which was scored: “-0.5” (clear mismatch), “0.5” (language mostly matches) or “1” (language clearly matches). The RPAD provides a total maximum score of nine.

##### Dose and timeliness of intervention delivery

Data related to the RESPOND modules chosen, dose delivered (number of intervention sessions, and total duration of intervention delivered), and timing of intervention contacts (time from ED discharge to the initial home visit, and subsequent telephone coaching calls), were recorded on the project database by the RESPOND clinicians following each intervention contact, and compared to the parameters set in the RESPOND RCT protocol (summarised in Fig. 1: planned activities).

##### Participant focus groups and RESPOND clinician interviews

The opinions of and experiences with the implementation fidelity components detailed above, from the perspectives of those participating in, as well as those delivering RESPOND, were captured qualitatively. Intervention participants’ perspectives were examined through focus groups at the completion of the intervention period. Following the intervention period, participants were contacted via telephone and invited to participate in a focus group, with a follow-up letter sent to individuals who agreed to participate. All focus groups were conducted by the lead researcher (RLM), using a discussion guide developed in consultation with the RESPOND investigator team. The guide included prompts to discuss opinions about program content, dose, delivery style, and delivery mode, as well as perceived benefits of and barriers and facilitators to participation.

The opinions and experiences of the RESPOND clinicians were identified through individual semi-structured audio-recorded interviews, following the intervention period. The interview discussion guide mirrored that of the focus groups to allow for comparison between the experiences of those delivering and receiving the program.

The lead researcher conducted the focus groups and interviews, and field notes were taken. All interviews and focus groups were audio-recorded and transcribed. Copies of the transcripts were sent to the participants to provide the opportunity to comment on accuracy.

#### Barriers and facilitators

Barriers and facilitators to implementing RESPOND were identified through the participant focus groups and clinician interviews as detailed above. In addition, clinicians routinely asked participants to identify barriers and facilitators to achieving RESPOND goals as part of the intervention sessions. These were recorded in the project database via ‘tick box’ categorical options.

##### Acceptability

Acceptability of RESPOND was determined using a purpose-designed questionnaire sent to all intervention participants on completion of the 6 month RESPOND program. The questionnaire comprised nine Likert-type five point scale questions (strongly agree to strongly disagree) exploring opinions related to key program components, and perceived benefits and satisfaction with participating in RESPOND. A further four questions explored participant opinions related to the mode of delivery (one face-to-face visit and telephone calls) and dose delivered, with categorical options to choose from.

#### Data analysis

A random selection of 10% of all audio-recorded intervention sessions were used to analyse clinician adherence. The lead researcher analysed the audio-recordings, in accordance with the purpose-designed analysis guide, and the RPAD coding manual (obtained on request from C.G. Shields [30]). A second researcher analysed 20% of the selected audio-recordings to determine inter-rater consistency and ensure rigour. Discrepancies were discussed until consensus was reached. An inter-rater discrepancy of <10% was considered acceptable. Descriptive statistics were used to summarise all quantitative data, using Stata version 14 [31].

Qualitative data were analysed by the lead researcher using deductive and inductive coding [32]. Coding was guided by the assumptions underlying the RESPOND program logic, and key components of the RESPOND program design: person-centredness, motivational interviewing, provision of education and community linkage, dose and timeliness of intervention delivery, perceived relevance and benefit of RESPOND, and barriers and facilitators to implementation. An inductive approach was used to code relevant features of the data beyond the pre-defined categories described above. Coding was validated by a second researcher who coded 10% of the transcripts selected at random to ensure rigour with difference being resolved by consensus. Coding was supported by NVivo version 11 [33]. Themes were identified from the codes and mapped to the Capability Opportunity Motivation – Behaviour (COM-B) model [34]. This model categorises behaviour (B) as the result of an individual’s capability (C); opportunity (O); and motivation (M), to perform the behaviour. The behaviours of interest for this evaluation were: (i) participation in the RESPOND program (intervention participants); and (ii) delivery of RESPOND (RESPOND clinicians). The themes and their categorisation in the COM-B model were reviewed by a second researcher and refined following discussion and consensus.

For each evaluation component, quantitative and qualitative data were synthesised at the interpretation and reporting level. Data were integrated through narrative, using a weaving approach, with qualitative and quantitative findings reported together on a component-by-component basis [35].

As the trial was conducted in two Australian States, it was possible that State-specific contextual variations could have influenced implementation of the program. An inter-site comparison was made to determine fidelity across sites using chi square tests for categorical data and t-tests for continuous data, with a *p* value of < 0.05 considered statistically significant.

## Results

**Fig. 2:**
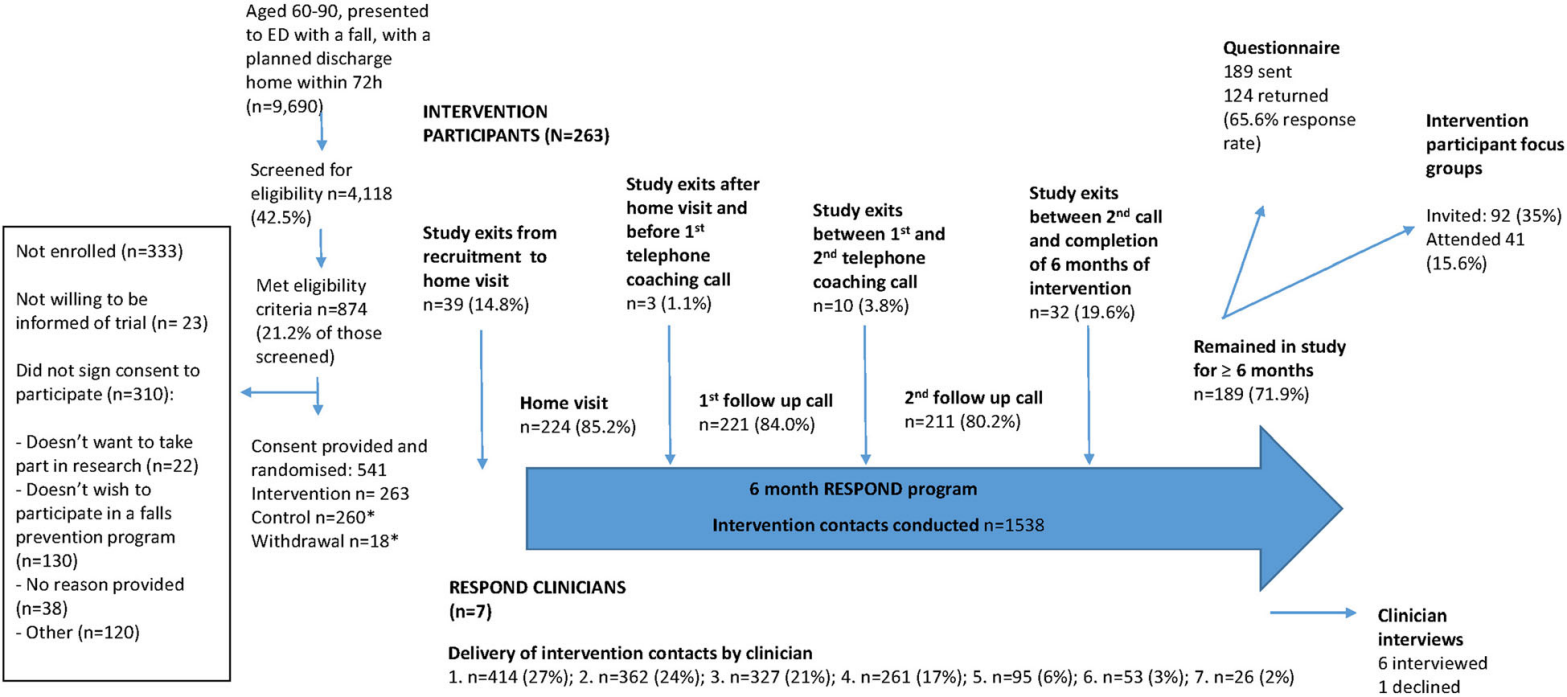
Participant flow. *Data from control participants, and those who withdrew from the study prior to completion, are not included in this process evaluation

Intervention participants were a mean (SD) age of 73 (8.4) years, with the majority (71%) of high socio-economic status. A large proportion (42%) of participants lived alone, and a further 36% were a high falls risk. Participant characteristics are presented in Table 1. A total of 224 (85%) of all participants randomised to the intervention participated in at least one intervention session. The seven RESPOND clinicians contributed various proportions of intervention delivery. Six clinicians participated in interviews, with one declining (clinician 6). Participant flow through the study is summarised in Fig. 2.

**Table 1:** Participant characteristics

RESPOND intervention participant characteristics	
Recruitment	*n* = 263
Female, n (%)	132 (50.2)
Age group, n (%)	
60–69	107 (40.7)
70–79	89 (33.8)
80–90	67 (25.5)
Socio-economic status^a^	
1st quartile	4 (1.5)
2nd quartile	22 (8.4)
3rd quartile	51 (19.4)
4th quartile	186 (70.7)
Home visit	*n* = 224
Lives alone, n (%)	93 (41.5)
Number of falls^b^, n (%)	
1 fall	135 (60.2)
2 falls	51 (22.8)
≥ 3 falls	38 (17.0)
Number of comorbidities^c^, n (%)	
None	53 (23.6)
1	55 (24.6)
2	53 (23.7)
≥ 3	63 (28.1)
Falls risk^d^	
Mild, n (%)	54 (24.1)
Moderate, n (%)	90 (40.2)
High, n (%)	80 (35.7)

^a^Socio-economic status was approximated using the Index of Relative Socio-Economic Advantage and Disadvantage (IRSAD) [36]. The 1st quartile (25th percentile) represents those with the most disadvantage, with the 4th quartile (100th percentile) representing those with the most advantage

^b^Number of falls in the last 12 months (including the index fall) was reported by participants as part of the Falls Risk for Older People – Community setting (FROP-Com) risk assessment tool

^c^ Number of comorbidities was reported by participants as part of the FROP-Com assessment. Defined as total number of diagnoses of: arthritis; any respiratory condition; Parkinson’s Disease; diabetes; dementia; peripheral neuropathy; any cardiac condition; stroke; any other neurological condition; lower limb amputation; osteoporosis; vestibular disorder; or lower limb joint replacement

^d^ Falls risk was determined from the FROP-Com total score (0–60): mild = 0–11; moderate = 12–18; high = 19–60 [25]

### Implementation fidelity

#### Reach

Over the study period, 9690 people aged 60–90 years presented to the two EDs with a fall, and had a planned discharge home within 72 h; of these, 4118 (43%) were screened for eligibility. The remainder either presented outside trial recruitment times or were discharged before recruitment could occur. Of those screened, 21% met all eligibility criteria. Of those eligible but not enrolled (*n* = 333), 39% did not want to participate in a falls prevention program, and 7% did not wish to be part of a research project (Fig. 2).

#### Intervention participant adherence

Better Strength and Balance was the most frequently selected module (*n* = 204; 91% of participants who received the intervention), followed by Better Bones (*n* = 148; 66%). Better Sleep and Better Eyesight were the least frequently chosen (*n* = 81; 36% and *n* = 72; 32% respectively). Participants chose a median (IQR) of 2 (2–3) modules over the intervention period. Five of the 224 participants who received at least one intervention session did not choose a module throughout their intervention period. Two of these five dropped out after their home visit, one dropped out after their first follow up coaching call, and one after their second call. The fifth participant was lost to follow up after six follow up coaching calls. Adherence to the program was defined as choosing at least one module, completing a minimum of three intervention sessions and setting at least one goal. A total of 195 of the 263 intervention participants (74%) met these three minimum requirements. Participants who chose Better Strength and Balance had the highest proportion of adherence 180 (88%); with similar proportions for Better Eyesight and Better Bones (*n* = 55, 76% and *n* = 111, 75% respectively). The lowest adherence was for those who chose Better Sleep (*n* = 41, 51%).

#### RESPOND clinician adherence

A total of 926 sessions (60% of all intervention contacts) were audio-recorded by the RESPOND clinicians. Ten percent (*n* = 93) of recordings were randomly selected for inclusion in the analysis. Overall, the RESPOND clinicians delivered the program in a person-centred manner, as indicated by the RPAD scores (median RPAD score 7; IQR: 6.5–7.5) (Table 2). Some aspects of participatory decision-making were exemplary, with evidence of the clinicians matching their language to the participants’ level of understanding in all of the analysed intervention contacts. The clinicians explained the issue, asked open ended questions, and checked their understanding of the participant’s point of view in over 90% of analysed audio-recordings. However, there was little evidence (4%) of the clinicians asking the participants if they had any questions (Table 2).

**Table 2:** Implementation fidelity

RESPOND program component	Median (IQR)	Protocol requirement	Protocol requirement met by those remaining in the study at the time point of interest, *n* (%)	% of total intervention cohort who met requirement (*n* = 263)
Intervention participants who received at least one intervention session (home visit) *n* = 224				
Number of intervention contacts (home visit plus telephone calls)	7 (5, 8)	1 home visit + 2 telephone calls	211 (94.2)	80.2
Total duration of direct intervention provided per participant (hours)	2.9 (2.1, 4)	≥ 10 h	0 (0)	0 (0)
Total duration of intervention (days)	171 (158, 178)	6 months (> 182 days)	38 (17.0)	14.5
Duration of home visit (minutes)	45 (30, 50)	≥ 45 mins	114 (50.9)	43.3
Days from ED discharge to home visit (days)	18 (12, 30)	≤14 days	85 (38.1)	32.3
Intervention participants who received at least 1 follow up coaching call *n* = 221				
Duration of each telephone contact (minutes)	20 (15, 25)	≥ 45 mins	2 (0.9)	0.8
Days from the home visit to the first coaching call (days)	14 (9, 17)	≤ 14 days	148 (67.0)	56.3
Intervention participants who received at least 2 follow up coaching calls *n* = 211				
Days from the first to the second coaching call (days)	21 (14, 30)	≤ 3 months (91 days)	207 (98.1)	78.7
Audio-recordings of intervention sessions *n* = 93				
RPAD 1) Clinician explains the clinical issue or nature of the decision	1 (1, 1)	Scored 1	92 (98.9)	
RPAD 2) Clinician discusses uncertainties associated with the situation	0.5 (0, 1)	Scored 1	43 (46.2)	
RPAD 3) Clarification of agreement with the management plan	1 (0.5, 1)	Scored 1	51 (54.8)	
RPAD 4) Examining barriers to follow-through with management plan	1 (1, 1)	Scored 1	78 (83.9)	
RPAD 5) Participant asks questions	0.5 (0.5, 0.5)	Scored 1	17 (18.3)	
RPAD 6) Clinician’s medical language matches participant	1 (1, 1)	Scored 1	93 (100)	
RPAD 7) Clinician asks, “any questions?”	0 (0, 0)	Scored 1	4 (4.3)	
RPAD 8) Clinician asks open ended questions	1 (1, 1)	Scored 1	87 (93.6)	
RPAD 9) Clinician checks their understanding	1 (1, 1)	Scored 1	88 (94.6)	
RPAD total score	7 (6.5, 7.5)	Scored 9	0 (0)	
Falls risk and management education provided		Yes	89 (95.7)	
Linkage to community falls prevention services provided		Yes	88 (94.6)	
Motivational interviewing: Open-ended questions		Yes	87 (93.6)	
Motivational interviewing: Affirmation		Yes	88 (94.6)	
Motivational interviewing: Reflection		Yes	80 (86.0)	
Motivational interviewing: Summary		Yes	79 (85.0)	

Qualitative data demonstrated that a person-centred, participatory decision-making approach was favoured by clinicians and participants:

“*When people set their own goals it’s often a lot more empowering and they’re often a lot more motivated to actually do them because they’ve come up with them themselves.*” (Clinician 1).

“[The RESPOND clinician] *encouraged you and sort of steered you in the right* [direction] *or gave you options… if someone tells me what to do I just ignore it.*” (Male participant, aged 68).

The clinicians implemented at least one motivational interviewing technique in the majority of intervention sessions (85–95%), with 71% (*n* = 66) of recorded contacts demonstrating evidence of all four OARS components (Table 2). The clinicians recognised that motivational interviewing techniques were a useful strategy for delivering behaviour change interventions:

*“I think motivational interviewing is really appropriate whenever you’re dealing with any kind of health care.”* (Clinician 1).

However, some clinicians found that this approach worked better with some participants than others:

*“Using* [motivational interviewing] *in the purer sense was difficult at times… There’s a couple of male* [RESPOND participants] *that come to mind who don’t want to have in-depth conversations. They really want a “yes/no”. Some people are used to a very prescriptive style of care.* ” (Clinician 5).

Falls prevention education was provided in most (96%) of the analysed intervention sessions (Table 2). The clinicians and participants recognised the benefits of providing education related to falls risk and associated management strategies:

*“I think bringing new ideas to them, new information, new education, that was also a key benefit, and a lot of people didn’t have a lot of this knowledge, and they were really grateful for that”*. (Clinician 7).

*“They* [RESPOND clinicians] *were informative… and explained them* [the RESPOND modules] *all very thoroughly”*. (Female participant, aged 62).

The clinicians linked participants with appropriate community services in 95% of analysed audio-recordings. The participants appreciated having an allocated clinician to facilitate this community linkage:

*“Before I had the fall I did strength training with* [community health centre]. *After the RESPOND clinician came to me see me, I said I wanted to go back to the exercise program, but if I’d just rung the exercise program and said I’d like to go back, I would have been on the waiting list for six months. I said ‘this is my goal, I’d like to go back to this exercise program’.* [My RESPOND clinician] *either phoned ... they did something, which meant that I was able to get in much quicker, and that was very helpful. And I’m still involved in that, and I intend to continue it”.* (Female participant, aged 67).

The unique role of the RESPOND clinician as the ‘missing link’ for providing coordinated falls prevention advice and support was recognised by clinicians at both sites:

*“When you actually look at it, I listen to that person for as long as they want to talk, and we make a plan of what to do next, and I encourage them. What other services do that? Very, very few”.* (Clinician 1).

*“I think it* [RESPOND] *does fill a gap….When someone turns up at the ED it’s unlikely that they’re going to get anywhere near the kind of information that RESPOND’s providing for them, and it’s a bit hit and miss with their GP as well just because they’re busy… the ongoing support* [provided by the RESPOND clinician] *over a period of time is really valuable for these people”.* (Clinician 7).

The participants expanded on this idea of RESPOND meeting a clinical need and suggested that it has particular value to those who live alone and/or are socially isolated:

*“She* [RESPOND clinician] *put me on to the right exercise program, she encouraged me, she helped me to get bits and pieces of furniture, lifting up the mats. I found her invaluable, plus having that support. When you live on your own, it’s a horrible experience”*. (Female participant, aged 79).

*“There must be other people, like me, that really don’t have anybody and you fill in a very important job*”. (Male participant, aged 74).

#### Dose and timeliness of intervention delivery

The majority of participants (80% of the total intervention cohort) received the minimum requirement of one home visit plus two follow up coaching calls. However, overall, the intervention was delivered at a lower dose than planned. Less than 1% achieved a telephone call that lasted 45 min or more (median 20 mins, IQR: 15, 25). No participants received the planned 10 h of intervention contact time with their RESPOND clinician, with a median total intervention time of 2.9 h (IQR: 2.1, 4) (Table 2).

However, the clinicians highlighted the importance of quality over quantity in terms of the dose of program delivered:

*“I’ve got another man who very seldom went over eight minutes in a call, and he just loved having the calls, and he was in a totally different place…in a positive way…at the end of that six months than the beginning”.* (Clinician 1).

The clinicians suggested that a higher dose was often associated with increased participant complexity:

*“Lower-functioning ones who needed more assistance and support, you could do a half-an-hour phone call with them”.* (Clinician 5).

Of those who had a home visit, less than half (38%) received this within the intended 2 weeks of ED discharge (median 18 days; IQR 12, 30) (Table 2). A further 85 (38%) received their first intervention session within 30 days, meaning that 76% of participants received their home visit within 1 month of ED discharge.

Clinicians cited complex health reasons as contributing to the delay in completing a home visit:

*“Perhaps all of the health issues weren’t immediately understood when they were seen in ED so sometimes that would mean re-presentations or it would mean later on they’d end up being admitted to rehab… or staying on in the hospital… or they’d gone to stay with family”.* (Clinician 1).

Despite the challenges of delivering an early intervention, the participants perceived value in receiving the RESPOND program during the vulnerable post-fall period:

“[RESPOND] *really helped in those first few weeks when you’re at home and you’re sort of thinking ‘oh my god, what have I done here?’ I just found that very reassuring. I was very impressed”.* (Female participant, aged 62).

Nearly all participants (98%) received their second coaching call within 3 months of the first call (Table 2). The clinicians perceived the frequency of intervention sessions as important for maintaining progress towards RESPOND goals:

*“In terms of frequency I think you need to stay in touch with them every two or three weeks otherwise they forget and it becomes strange to talk something that you have discussed at the last phone call”.* (Clinician 5).

Inter-site consistency was high with no statistically significant differences between sites for program dose, timeliness, or delivery of key program components.

### Barriers and facilitators

#### Capability

The main ‘capability’ barrier to participation in RESPOND was participants’ complex health issues taking priority and/or limiting the participant’s physical capacity to take part (Table 3). Complex health issues fell into the following main categories: recent surgery; an exacerbation of an existing condition; or new medical diagnosis and associated treatment. Conversely, medical clearance to exercise (physical capacity to participate in falls prevention exercises following fall-related musculoskeletal injury, as judged by the participant’s GP or other medical professional) was stated as a facilitator for participants to engage in RESPOND activities. Increased awareness of falls risk factors and their associated management strategies, resulting from the educational component of RESPOND, was also reported as a key facilitator to participants’ capability to engage in RESPOND.

*“*[RESPOND is] *worth doing from the point of view that they make you aware of the reasons why you have a fall… I think the information was beneficial…it made me change my lifestyle”.* (Female participant, aged 62).

For the clinicians, lack of prior knowledge or training for delivering certain RESPOND components was viewed as a barrier to delivering RESPOND. The clinicians considered prior relevant experience as a facilitator to their perceived capacity to deliver RESPOND, with a bias towards modules that correlated most closely with their professional background:

*“I skew more to strength and balance and bones, because it’s something I know a lot more about than, say, vision or sleep”.* (Clinician 7).

**Table 3:** Barriers and facilitators to participation in and delivery of RESPOND, mapped to the COM-B Framework

		Participant Behaviour = participation in RESPOND Theme	Clinician Behaviour = delivery of RESPOND Theme
Capability: physical and psychological capacity to engage in the behaviour	Barrier	• Complex health situation^CR^	• Lack of prior knowledge or training for delivery of specific RESPOND components^I^
	Facilitator	• Increased awareness of falls risk factors and their management strategies^FG^ • Medical clearance to commence exercise program^CR^	• Prior work experience or training in certain aspects of RESPOND^I^
Opportunity: external factors that make the behaviour possible	Barrier	• Complex social situations^CR^ • Insufficient time^CR^ • RESPOND recommendations not supported by participant’s primary healthcare provider^CR, FG^	• Participants’ competing priorities (health and social)^I^ • Participants’ lack of perceived relevance^I^
	Facilitator	• Access to transport^CR^ • Adequate time^CR^ • Financially viable^CR^ • Services readily available^CR^ • Supportive primary healthcare provider^FG^	• RESPOND education pamphlets as basis for intervention sessions^I^ • Participants’ perceived relevance^I^
Motivation: brain processes that direct behaviour, such as decision-making, habitual processes and emotional responses	Barrier	• Lack of perceived relevance^CR, FG^	• Clinical decision-making within the constraints of the RCT^I^
	Facilitator	• Support from RESPOND clinician^CR, FG^ • Perceived personal relevance^CR, FG^ • Positively-framed health messages^FG^ • Participatory decision-making^FG^	• Peer support^I^ • Person-centred approach^I^ • Rapport with participant^I^ • Positively-framed health messages^I^

Data source: *CR* Clinician records, *FG* Focus group (participants), *I* Interview (clinicians). This table is based on the COM-B framework [34]

#### Opportunity

The external factor that was perceived as the greatest barrier to participating was complex social issues. This most frequently related to carer commitments (caring for a spouse, or grandchildren); breakdown of personal relationships; social engagements; or travel. Lack of time was an additional barrier for some, most commonly due to work commitments. Some participants also reported their primary healthcare provider sometimes posed a barrier to completing agreed actions in order to achieve RESPOND goals:

*“My doctor wouldn’t give me a referral to have the vitamin D checked. He said it was an overtreatment and unnecessary”.* (Female participant, aged 71).

The participants’ other health and social issues were also identified as key ‘opportunity’ challenges for the clinicians delivering RESPOND, because participants’ priorities were elsewhere:

*“A lot of comorbidities makes it essential but difficult”.* (Clinician 7).

*“She* [RESPOND intervention participant] *had all this other emotional stuff – family issues – going on that were a higher priority* [than RESPOND] *to deal with”.* (Clinician 5).

In some instances, RESPOND appeared less relevant for participants and engaging them in the program posed a challenge for clinicians:

*“Those who came through with a really severe health event, or an accident… and don’t even classify it as a fall, it was harder to see a link between what we’re offering and what’s happening in their life. There was not so much relevance there.”* (Clinician 7).

Key facilitators for participants included adequate access to transport, affordable and accessible services, and having sufficient time for the intervention sessions and to address RESPOND goals.

The clinicians identified the RESPOND pamphlets as facilitating the delivery of RESPOND by providing a prompt and focus for the intervention sessions:

*“To leave them with people so that they could look at them and then ask them, ‘Had they looked at them since you’d spoken?’, ‘Was there anything else that came up out of them?’, and as a memory jogger. Sometimes they used them as a cue when they went to their GP to cover some element of whichever module was involved. So, yeah, I found them quite useful”*. (Clinician 5).

The clinicians found that participants were more engaged in the program if they perceived RESPOND to be personally relevant:

*“Some of those people* [RESPOND participants] *would definitely relate* [to the RESPOND modules] *if they were looking at their health and general wellbeing and going ‘yeah, I notice that my balance has been getting a little bit worse in the last few weeks’. These are the words and the language that you could usually pick up from the conversation and go, great, I think there’s going to be some perceived relevance and some acceptance here”.* (Clinician 7).

#### Motivation

Lack of perceived relevance was a key motivational barrier to participation for some participants:

*“I think it* [RESPOND] *is more for people that have a ‘proper’ fall”.* (Male participant, aged 84).

Conversely, perceiving the RESPOND modules to be personally relevant, was a motivating factor for many:

*“Once you have one fall the chances of you having another fall are high. So it* [RESPOND] *really made me aware of that…I was off to exercise”.* (Female participant, aged 60).

For the clinicians who were used to having a broad repertoire of patient management options in real-world clinical situations, performing clinical decision-making and treatment within the constraints of an RCT sometimes posed a challenge to delivering RESPOND.

*“The thing is when you’ve got fixed strategies like we do in our modules, to let a person take their choices and be the one guiding their choices… is such a difficult thing to do”*. (Clinician 7).

*“Having the four specific modules that we were to stick to was really tricky”.* (Clinician 1).

Participants considered decision-making support from the RESPOND clinicians to be a main motivational factor. This included problem-solving identified barriers to participation, practical suggestions for navigating the healthcare system, and adapting strategies to achieve RESPOND goals. Words used by the participants to describe their clinicians include: *“supportive”; “friendly”; “caring”; “approachable”; “encouraging”; “motivating”; “uplifting”.* In a similar theme, the clinicians identified their rapport with the participants as a factor that facilitated their delivery of the program.

Health messages delivered in a positively-framed manner were facilitating factors for both participants and clinicians. Specifically, participants and clinicians identified the RESPOND education pamphlets and their “Be Your Best” slogan as non-confrontational and motivating:

*“There’s nothing in here to say you had a fall…it’s just ‘be your best’ …and happy, older person on the front... and it’s health education. I think this is excellent.”* (Male participant, aged 76).

*“I think it’s good – especially for those patients who are very fall-phobic.”* (Clinician 1).

### Acceptability

**Fig. 3:**
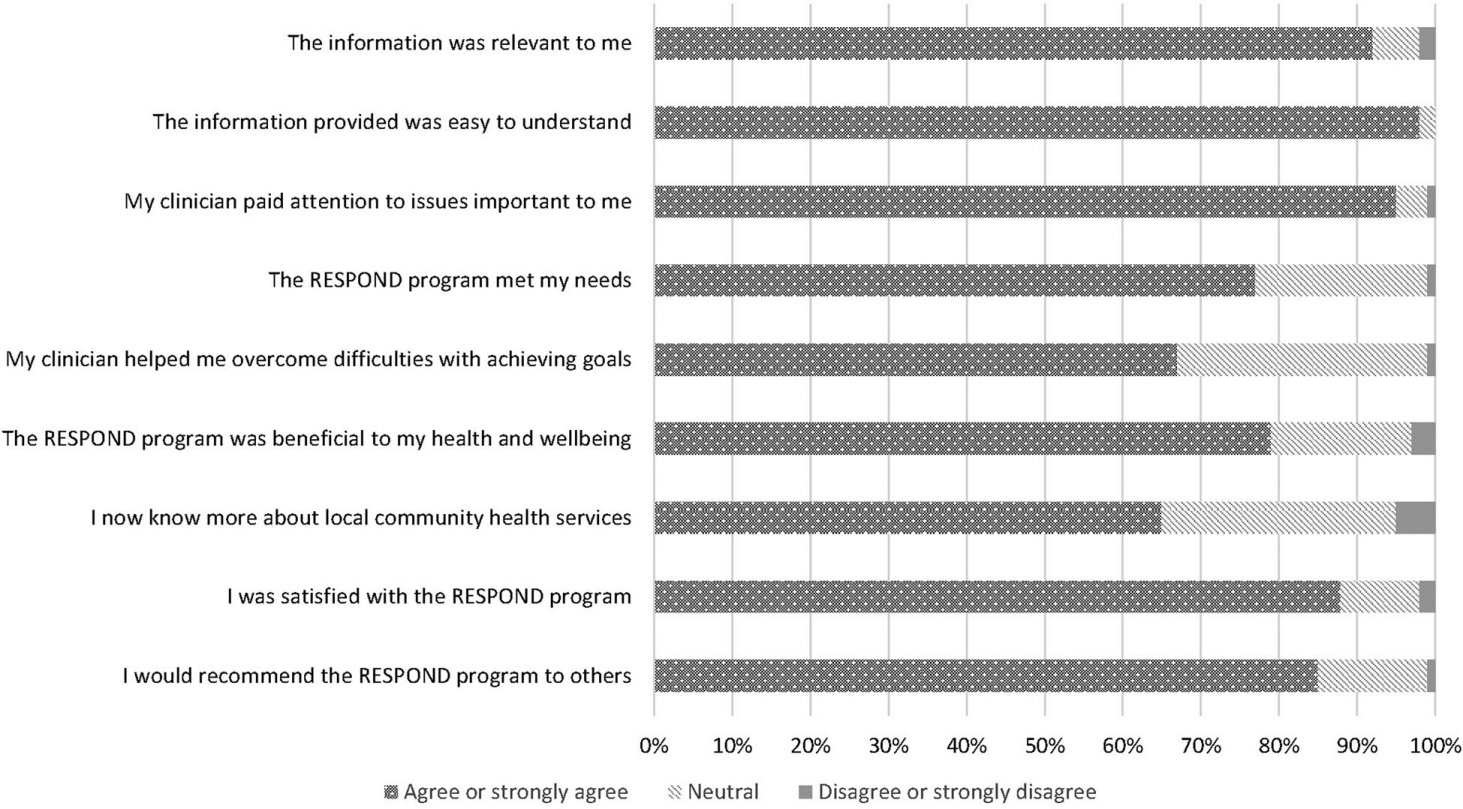
Participant acceptability and satisfaction from questionnaire results

Over half of the participants who received the intervention (*n* = 124, 55%) completed the post-intervention questionnaire. The majority of respondents perceived the program to be acceptable and were satisfied with the program (87%) (Fig. 3). Half (51%) were satisfied with the mode of delivery (one face-to-face home visit with subsequent telephone calls) with 23% preferring to only talk over the phone, and 11% preferring to only have face-to-face meetings with their RESPOND clinician. A further 9% preferred other modes of communication, such as email, and the remaining 6% left this question blank. Mixed opinions regarding mode of delivery were also evident in the focus groups, however, the majority were happy with the RESPOND format:

*“I think one visit’s enough… I loved the phone calls much better.”* (Female participant, aged 60).

The clinicians found the telephone calls to be flexible and convenient:

*“Some of them would say ‘here’s my mobile number, call me on my mobile, I’ll be out and about but I’ll answer it’… so it was very convenient for them.”* (Clinician 1).

However, the clinicians also valued the face-to-face session in terms of rapport building:

*“I feel like when you’ve spent more time with them in the house they’re more likely to relax and chat to you longer on the phone at the subsequent follow-ups because you’ve got a little rapport.”* (Clinician 7).

A similar sentiment was expressed by the participants:

*“I like the phone calls, but it was also nice to have the initial face-to-face and meet the person, that’s just a nice way to communicate with somebody.”* (Female participant, aged 62).

Of the participants who preferred the home visits over the phone calls, social interaction was commonly stated as the main reason:

*“I personally like the visits… but that’s probably because I am on my own so much.”* (Male participant, aged 74).

The total number of telephone calls was considered acceptable, with 89% of participant questionnaire respondents stating that they had just the right amount of calls and 85% felt the program length of 6 months was just right.

## Discussion

This is the first comprehensive process evaluation to be performed in parallel with an RCT of a falls prevention program that significantly reduced the rate of falls and fractures for older people who have presented to an ED with a fall. Our evaluation showed that RESPOND was effective at a substantially lower dose than intended, and the program content and style was acceptable to participants and clinicians. This evaluation suggests that the critical success factors are: i) the delivery style - delivering positively framed health messages in a person-centred manner, using motivational interviewing techniques; ii) the program content - the provision of consistent support, targeted education, and coordination of community services; and iii) timely intervention - the first session being conducted within 1 month of ED discharge.

An important aspect of person-centred care is participatory or shared decision-making. This involves people making informed decisions based on facts as well as their personal values and preferences [37]. The RESPOND clinicians delivered the program in a person-centred manner, as evidenced by the overall RPAD scores. Importantly, this style of program delivery was preferred by RESPOND participants and clinicians.

In addition to participatory decision-making, motivational interviewing is a well-established method for accomplishing person-centred care [38]. However, motivational interviewing has only been used to a limited extent with older adults [27]. The current evaluation demonstrated that over 70% of analysed audio-recordings of RESPOND intervention sessions had evidence of the clinicians using all four key motivational interviewing ‘OARS’ skills; this may have contributed to the positive RCT results. Similarly, a recent study found that provision of motivational interviewing was associated with older adults’ adherence to a falls prevention exercise program at 1 year [39].

RESPOND education and the accompanying module pamphlets emphasised maximising independence and functional capabilities to allow people to “Be Your Best”, rather than focusing on reducing falls and the associated negative connotations [40]. This was well received by the RESPOND participants, and the clinicians found the positively-framed messages facilitated their delivery of the program. This finding is consistent with the literature. A meta-analysis found that ‘gain-framed’ messages appear to be more effective than ‘loss-framed’ messages in promoting prevention behaviours [41]. This is supported by a recent study that concluded that older adults prefer falls prevention information to be delivered in a positive tone [42]. In contrast, Haines et al. (2014) suggested that explicitly discussing falls and falls risks is required to overcome the “better for others than me” attitude to falls prevention activities [43]. However, only 36% of their study participants had experienced a fall in the last 12 months, compared with 100% of RESPOND participants, which may account for differences in the perceived relevance and benefit of engaging in falls prevention activities.

The importance of education in reducing falls has been previously demonstrated [44]. Importantly, RESPOND participants mostly found the information provided to be personally relevant, which has been found to be more motivational for engaging in fall prevention activity [45]. An additional finding from our study was the importance of the relationship built between the participant and the education provider - the clinician. The concept of preventive information being provided with empathy and time to listen has been shown to foster motivation and engagement in recommended activities [42]. The rapport established between the RESPOND clinicians and the participants emerged as a factor that facilitated the delivery of the program, and motivated the participants. This support for the participants for the first 6 months following an ED presentation for a fall appears to address a clear gap in existing falls prevention services. This may be especially pertinent for those living alone or socially isolated. Prior studies highlight the importance of social support for maintaining health and function for older adults [46–48].

The RESPOND intervention was not as timely as planned (the initial home visit was intended to be conducted within 2 weeks of ED discharge). However, despite not achieving trial protocol, most participants were seen within 1 month of ED discharge. Delivery of the intervention within 1 month of the index fall appears to differentiate successful programs from others [7]. A Dutch RCT cited the time lag for intervention as a reason for the ineffectiveness of the program, with medical and occupational therapy assessments taking place five and 10 weeks after baseline, respectively [17]. In contrast, a successful UK trial delivered services within 1 month of ED discharge [16]. The main reasons identified for the delay in delivering the initial RESPOND intervention session were the participants’ complex health and social issues acting as competing priorities. These factors should be considered when planning appropriate timing of intervention sessions.

RESPOND was effective at reducing falls and fractures at a lower dose than anticipated (median of 3 h, compared with the planned 10 h), thereby requiring fewer resources. The concept of ‘quality over quantity’ was cited as a reason for brief intervention sessions. Despite the relatively short contact duration, the median number of intervention sessions was seven per participant, exceeding the minimum of three contacts stated in the protocol. This suggests that frequency may be more beneficial than duration of intervention contacts. This was supported by the clinicians’ perceptions that regular clinician contact maintained participant progress towards goals. However, the delivery of a substantially lower dose of intervention than planned may be a reason for the lack of impact on falls injuries or hospitalisations. Further information is required to better understand the program dose or other factors required to support reduction in fall injuries and hospitalisation outcomes.

Refining the clinician training program is recommended. RPAD item scores indicated a need for further training related to consistently asking the participant if they have any questions. Interview data showed that clinicians were more confident delivering aspects of RESPOND that they had prior knowledge or experience with. This suggests that RESPOND clinician training and resources may need to be tailored to account for individual expertise and professional backgrounds.

A further suggestion for future implementation is to allow increased flexibility with the mode of program delivery. The home visits were valued by clinicians and participants, particularly those who live alone or are socially isolated, and some participants may benefit from additional face-to-face sessions. Similarly, addition of alternative methods of communication, such as email or text messaging, may improve engagement for some.

This evaluation has a number of methodological strengths. The use of a mixed methods approach, with pre-specified data collected alongside the multi-centre RCT, allows for a rich understanding of the RESPOND trial results to be generated. Our evaluation of program fidelity through analysis of audio-recordings reduced the risk of bias associated with clinician- or participant-reported data alone.

We also acknowledge the study limitations. While program acceptability was high among participants who returned the participant questionnaire, the opinions of those who exited the intervention prior to 6 months or chose not to complete the questionnaire could not be captured. Similarly, those who chose to attend the focus groups are unlikely to be representative of those who declined to participate, or exited the study prior to completion. However, this was somewhat mitigated through the additional data related to barriers and facilitators recorded following each intervention session. A further limitation is that despite participant adherence being high, as per our definition, we do not have data related to whether participants acted on recommendations made by their clinicians, and whether their goals were met. A separate paper will augment this study by reporting: i) participation in falls prevention strategies, comparing the RESPOND RCT intervention and control groups; and 2) sub-group analyses of intervention participants to determine who RESPOND is most effective for, as described in the RESPOND program evaluation protocol [24].

## Conclusions

This process evaluation found that RESPOND was delivered in a timely and person-centred manner, with positively-framed, personally relevant health messages aiding participant engagement. These appear to be the critical success factors for the significant reduction in the rate of falls and fractures. Participants’ complex health and social issues pose the greatest challenge to implementation fidelity. A lower than planned dose delivered may account for the lack effect on fall injuries or hospitalisation. The results of this process evaluation can provide guidance to researchers, clinicians, and policy makers on implementation of RESPOND, or similar programs, in other clinical settings.

## Data Availability

The datasets generated and/or analysed during the current study are available from the corresponding author on reasonable request.

## Abbreviations

COM-B: Capability, opportunity, motivation – behaviour (behaviour change framework)
ED: Emergency department
FROP-Com: Falls Risk for Older People – Community setting (falls risk assessment tool)
GP: General practitioner
IQR: Interquartile range
MI: Motivational interviewing
OARS: Open-ended questions, affirmations, reflections, summaries (motivational interviewing skills)
RCT: Randomised controlled trial
RPAD: Rochester Participatory Decision-Making Scale
