# Correction: A mixed methods process evaluation of a person-centred falls prevention program

**DOI:** 10.1186/s12913-023-09743-7

**Published:** 2023-06-27

**Authors:** Rebecca L. Morris, Keith D. Hill, Ilana N. Ackerman, Darshini Ayton, Glenn Arendts, Caroline Brand, Peter Cameron, Christopher D. Etherton-Beer, Leon Flicker, Anne-Marie Hill, Peter Hunter, Judy A. Lowthian, Renata Morello, Samuel R. Nyman, Julie Redfern, De Villiers Smit, Anna L. Barker

**Affiliations:** 1 School of Public Health and Preventive Medicine, Monash University, Melbourne, Australia; 2 Rehabilitation, Ageing and Independent Living (RAIL) Research Centre, Monash University, Melbourne, Australia; 3 School of Physiotherapy and Exercise Science, Curtin University, Perth, Australia; 4 University of Western Australia, Perth, Australia; 5 Harry Perkins Institute of Medical Research, Perth, Australia; 6 Melbourne EpiCentre, University of Melbourne and Melbourne Health, Melbourne, Australia; 7 Alfred Health, Melbourne, Australia; 8 Royal Perth Hospital, Perth, Australia; 9 Bolton Clarke Research Institute, Bolton Clarke, Melbourne, Australia; 10 Department of Psychology and Ageing & Dementia Research Centre, now at Department of Medical Science and Public Health, Bournemouth University, Dorset, UK; 11 Westmead Applied Research Centre, Faculty of Medicine and Health, the George Institute for Global Health, University of Sydney, Sydney, Australia

**Correction**: ***BMC Health Serv Res*** 19, **906 (2019)**

10.1186/s12913-019-4614-z

Following publication of the original article [1], an error was identified in the “Conclusion” section of the abstract.

The sentence “RESPOND was person-centred and reduced falls and fractures at a substantially lower dose, using fewer resources, than anticipated.” should be replaced with “RESPOND, which was previously shown to reduce falls and fractures in the main trial analysis, was found to be delivered in a timely and person-centred manner, at a substantially lower dose, using fewer resources than anticipated.”

The original article [1] has been corrected.
